# An Overview of Novel Dietary Supplements and Food Ingredients in Patients with Metabolic Syndrome and Non-Alcoholic Fatty Liver Disease

**DOI:** 10.3390/molecules23040877

**Published:** 2018-04-11

**Authors:** Priscila Silva Figueiredo, Aline Carla Inada, Melina Ribeiro Fernandes, Daniela Granja Arakaki, Karine de Cássia Freitas, Rita de Cássia Avellaneda Guimarães, Valter Aragão do Nascimento, Priscila Aiko Hiane

**Affiliations:** 1 Post Graduate Program in Health and Development in the Central-West Region, Federal University of Mato Grosso do Sul, Campo Grande 79070900, Brazil; inadaaline@gmail.com (A.C.I.); fernandesrmelina@gmail.com (M.R.F.); daniarakaki@gmail.com (D.G.A); kcfreitas@gmail.com (K.d.C.F.); rita.guimaraes@ufms.br (R.d.C.A.G.); aragao60@hotmail.com (V.A.d.N); priscila.hiane@ufms.br (P.A.H.); 2 Optics and Photonics Group, Institute of Physics, Federal University of Mato Grosso do Sul, 549, Campo Grande 79070900, Brazil

**Keywords:** obesity, inflammation, nutraceuticals, natural products, polyunsaturated fatty acids

## Abstract

Metabolic syndrome (MetS) is characterized by interconnected factors related to metabolic disturbances, and is directly related to the occurrence of some diseases such as cardiovascular diseases and type 2 diabetes. MetS is described as one or both of insulin resistance and visceral adiposity, considered the initial causes of abnormalities that include hyperglycemia, elevated blood pressure, dyslipidemia, elevated inflammatory markers, and prothrombotic state, as well as polycystic ovarian syndrome in women. Other than in MetS, visceral adiposity and the pro-inflammatory state are also key in the development of non-alcoholic fatty liver disease (NAFLD), which is the most prevalent chronic liver disease in modern society. Both MetS and NAFLD are related to diet and lifestyle, and their treatment may be influenced by dietary pattern changes and the use of certain dietary supplements. This study aimed to review the role of food ingredients and supplements in the management of MetS and NAFLD specifically in human clinical trials. Moreover, bioactive compounds and polyunsaturated fatty acids (PUFAs) may be used as strategies for preventing the onset of and treatment of metabolic disorders, such as MetS and NAFLD, improving the inflammatory state and other comorbidities, such as obesity, dyslipidemias, and cardiovascular diseases (CVD).

## 1. Introduction

Metabolic Syndrome (MetS) represents a major clinical public health challenge worldwide given current urbanization, excessive energy intake, increasing obesity, and sedentary life habits [1]. MetS is an aggregation of interconnected metabolic factors that appear to directly develop into a cardiovascular disease (CVD), increasing the risk of the development of type 2 diabetes mellitus (DM2) [2,3]. 

Several groups of specialists have sought to establish simple criteria to identify in clinical practice patients who manifest the multiple components of MetS. In general, such criteria include a combination of underlying risk factors and metabolic factors, with different studies varying in the specific elements [2,4,5,6,7,8,9,10]. The consensus definition of MetS has been the global reference for its clinical diagnosis since its publication in 2009 [11]. 

The agreed-upon definition for MetS is the presentation of one or both of insulin resistance (IR) and visceral adiposity [12], which progress to be the initial cause of some abnormalities that include hyperglycemia, elevated blood pressure, and dyslipidemia, which is elevated triglyceride levels and reduced levels of high-density lipoprotein cholesterol (HDL-C), as well as elevated inflammatory markers, a prothrombotic state, and polycystic ovarian syndrome [13], which covers insulin resistance, oxidative stress, and an inflammatory state.

The IR process involves a decrease in the glucose disposition in the peripheral tissues, an overproduction of glucose by the liver, functional damage to pancreatic β-cells, and a decrease in the mass of these β-cells [14]. The cause of the latter is still under investigation, however, the available evidence suggests that hyperglycemia induces apoptotic cell death [15]. In the state of exacerbated consumption of calories that exceeds metabolic energy demand, saturated fatty acid (SFA) and glucose levels rise in blood, where they are then converted into triacyclglycerols (TG). Then, TGs are stored in the adipose tissue of visceral fat. Adipocytes release free fatty acids (FFAs) and inflammatory mediators like IL-1, IL-6, and TNF-α that can promote IR [16], since this effect is observed in parallel with low plasma levels of insulin, a hormone that prevents lipolysis. Under IR conditions, the counterregulatory effect of insulin does not occur and lipolysis in adipose tissue increases circulating levels of non-esterified fatty acids (NEFA) [13]. 

Additionally, a reduction in the activation of phosphotidylinositol-3-kinase (PI3k) occurs, as well as other proteins involved in the normal insulin signaling process, such as protein kinase B (Akt). These damage events appear to be related to the mediation of proteins that activate inflammatory pathways, such as the c-Jun NH2-terminal kinase (JNK), kinase IκB (Iκκβ), and PKCθ [17,18,19,20]. NFκB is a gene transcription factor that alters insulin signaling. After stimulation, Iκκβ is phosphorylated, leading to the translocation of NFκB to the cell nucleus and subsequent activation of proinflammatory cytokine genes, such as TNF-α, IL-6, and IL–1β [21,22,23]. The NFkB signaling pathway increases the generation of reactive oxidative species (ROS), creating a vicious cycle. Other important receptors in the activation of the immune system are then activated. In this case, the Toll-like receptors (TLRs) are generated, and particularly Toll-like receptor 4 (TLR4), which is associated with increased expression of inflammatory cytokines and other stimulatory molecules via the NFκB signaling pathway [24,25].

Adipocytes liberate monocyte chemoattractant protein-1 (MCP-1), which attracts macrophages and generates local inflammation that releases further cytokines. Adipocytes also contribute to IR and hypertension through the production of angiotensin and aldosterone. FFAs in the plasma are transported by the liver and packed into TG-rich VLDL, reducing the levels of high-density lipoprotein (HDL) and causing a HDL dysfunction. This reduces reverse cholesterol transport (RCT) and increases oxidized low-density lipoprotein (oxLDL) and MCP-1. Dysfunctional HDL modulates T-cells by inhibiting T-regulatory cell (Treg) and stimulating proinflammatory Th1 and Th17 cell production [16]. Notably, cardiovascular disorders are also mediated by the IR process through the MAPK-dependent pathway due to compensatory hyperinsulinemia that leads to a decrease in nitric oxide (NO) in the vessel and a greater secretion of endothelin-1 [26,27]. This increased secretion of endothelin-1 is reflected in vasoconstriction that leads to a decrease in glucose absorption and blood pressure [28].

Proinflammatory adipokines and NEFA derived from adipose tissue also damage the liver: they increase gluconeogenesis, preventing the inhibitory effect of insulin in this metabolic pathway, and stimulating lipogenesis [29]. Increased NEFA supply and lipogenesis in the liver lead to non-alcoholic fatty liver disease (NAFLD) and increased synthesis and release of low-density lipoprotein (VLDL), favoring the atherogenesis process [30]. Obese individuals have high plasma concentrations of cholesterol, VLDL-triacylglycerols, VLDL-ApoB, and LDL-ApoB, as well as hyperlipidemia and glucose intolerance [31].

NAFLD is common in Western countries, usually associated with the main features of metabolic syndrome, such as obesity, insulin resistance, and hyperlipidemia [32]. In addition to metabolic syndrome features, cellular senescence may lead to hepatic steatosis [33]. This liver disorder is the main cause of chronic liver disease, affecting 20% to 30% of the world’s adult population, characterized by a buildup of fat greater than 5% to 10% of the cell weight, mainly in the form of triglycerides in the cytoplasm of hepatocytes, determined histologically or by imaging [34,35]. Moreover, a portion of patients with NAFLD present liver cell injury and inflammation concomitant with excessive fat accumulation (steatohepatitis), which is referred to as nonalcoholic steatohepatitis (NASH) [36]. The pathogenesis of NAFLD are not completely understood and due to the fact it seems to be multi-factorial [37,38], some therapeutic strategies are used to treat patients with NAFLD and the most common pharmacological approaches include insulin-sensitizers like thiazolidinediones, such as pioglitazone [39], and lipid-lowering drugs, pentoxifylline and angiotensin receptor blockers [40,41]. Other studies emphasize the possibility that epigenetic manipulation through metabolic pathways may be a promising strategy to retard the progression of NAFLD [36,42]. Nonetheless, due to the complexity pathogenesis of NAFLD, there is a lack of consensus of a specific pharmacological strategy [40].

Regarding that NAFLD is associated with the most common features of MetS, strategies to reduce the onset and progression of MetS and their related pathologies are of interest [43]. Some lifestyle interventions that result in weight loss have benefits, since, from a metabolic point of view, physical activity can lower triacylglycerol (TG) levels via the activation of lipoprotein lipase (LPL), which hydrolyzes very low-density lipoprotein (VLDL) and then lowers TG levels. Conversely, inactivity leads to loss of skeletal muscle LPL, causing a shift from fatty acid to glucose oxidation, leading to a redistribution of TG to the heart and liver, and thus increasing TG in these tissues, which is conducive to IR [16]. Additionally, weight reduction alone can improve insulin sensitivity by reducing levels of TG. TG levels can be lowered 20–30% with weight loss [44]. 

MetS is associated with a greater risk of atherosclerosis and nonatherosclerosis [45]. Usual treatment involves a combination of lifestyle changes and the use of pharmacological therapy aimed at reducing or controlling CVD. The most common medicines used to control CVD include management of dyslipidemia with statins, decreasing prothrombotic risk with antiplatelet drugs, and the use of insulin sensitizers to decrease the risk of diabetes. However, no single drug therapy exists for MetS, and the pharmacotherapy for the associated comorbidities necessitate the prolonged use of multiple medications, which is challenging for patients due to polypharmacy and reduced compliance [46]. In addition, NAFLD is becoming a serious global health problem, accounting for the leading cause of liver disease. To date, no drugs have been approved for the treatment of NAFLD, and the major clinical recommendation for the initial step is lifestyle modification [47].

Lifestyle change is imperative in the management of MetS and its comorbidities. One essential strategy is an average weight reduction of 7–10% in baseline body weight over a period of 6–12 months, reducing caloric intake by 500–1000 calories/day [46], and engaging in physical activity for at least 150 min per week [48,49]. Dietary modification, including a 25–35% daily reduction in fats, and lowering intake of SFA, trans fatty acids, cholesterol, sodium, and refined carbohydrates, is associated with regulating other MetS risk factors like dyslipidemia, hyperglycemia and hypertension [46].

Actual evidence highlights some quality diets, such as the Mediterranean diet, the Nordic diet, and Dietary Approaches to Stop Hypertension (DASH) diet, to protect against MetS or to improve the MetS phenotype [50,51,52]. Interest in the use of natural compounds to control the risk and progression of MetS is also increasing. Dietary supplements that provide health benefits in addition to basic nutritional value are designated nutraceuticals, which include spices, herbs, essential oils, unsaturated fatty acids, and some natural compounds derived from plant extracts [46]. 

These nutraceuticals generally have high concentrations of phytochemicals, and/or mono- and polyunsaturated fatty acids, antioxidant vitamins, fibers, and minerals, which are related to the protective effects connected to these diets [53]. The presence of natural compounds, like phenolic phytochemicals, seem to provide metabolic benefits with respect to the major MetS disorders, including the anti-inflammatory, hypoglycemic, hypolipidemic, hypotensive, anti-atherosclerotic, anti-thrombotic, hepatoprotective, and hypocholesterolemic effects [54,55]. These molecules appear to regulate the expression of genes mostly involved in *de novo* lipogenesis and fatty acid oxidation, contributing to their lipid-lowering effect in the liver [56].

To date, studies have focused on the development of innovative therapeutic targets from dietary supplements and functional foods as an alternative remedy. However, consistently evaluating mechanisms of action and bioactive compounds that can systematically explain the effects of natural products on MetS and NAFLD, and especially their consequences, has posed a specific challenge [57,58].

Although nutraceuticals have been associated with potential benefits in the management of MetS and its comorbidities, and especially NAFLD, their long-term effect on the outcomes of the risk factors and long-term compliance is unknown. Furthermore, most of the studies have been focused in animal models, being that human studies are still scarce on the literature. Considering that metabolic differences exist between human and animals and diet the can varyingly influence the metabolic state and inflammation, we describe the effects of these dietary supplements and food ingredients specifically in clinical trials of adults with MetS and, at least in parts, in in vitro human cells, listing molecular mechanisms that may be involved in these metabolic effects.

## 2. Dietary Supplement and Functional Food: Alternative Nutrition Therapies

Dietary supplements derived from natural products, such as plants, spices, and herbs, with a variety of bioactive compounds, may beneficially affect metabolic parameters in adults with MetS and NAFLD. The beneficial effects of some isolated bioactive compounds, such as polyphenolic compounds like curcumin [59,60,61,62,63], resveratrol [64,65,66,67,68], and quercetin [69,70,71] on the metabolic parameters in these patients have been reported. 

Moreover, similar effects on metabolism were observed with other natural products, such as grape [72,73,74], green tea [75,76,77,78,79], orange juice [80,81,82,83], hibiscus [84,85,86], *Aloe vera* [87], *Wild bitter* [88] and berberine or barberry [89,90]. In turn, these food ingredients have been related to health promotion, chronic disease prevention, and adjunctive therapy in individuals with MetS and NAFLD. In addition to the positive impacts on metabolic disturbance in humans, synthetic supplements like vitamins, for instance vitamins D [91,92,93,94,95,96,97,98,99] and E [100,101,102,103], and polyunsaturated fatty acids (PUFAs), have been widely studied to contribute to the management of the chronic disease burden in human clinical trials. 

## 3. Bioactive Compounds: Polyphenolic Compound

### 3.1. Curcumin

Some studies in humans have shown that curcumin, an orange-yellow pigment extracted from turmeric, has a polyphenolic structure denominated curcuminoid with antioxidant and anti-inflammatory activities [59,60,61,63]. Inflammation and oxidative stress are key contributors to MetS and NAFLD. Curcumin (500 mg twice per day) and piperine (5 mg added to 500 mg of curcumin), improved oral curcumin bioavailability, and after eight weeks, were able to reduce serum levels of pro-inflammatory cytokines and adipokines, such as TNF-α, IL-6, IL-1β, and MCP-1, increased adiponectin, and reduced leptin levels between group-comparison in a post-hoc analysis of a randomized controlled trial in both men and women diagnosed with MetS (*n* = 59) [60,61]. 

In addition to these data, lipid-modifying effects were observed in patients with MetS (*n* = 59) in a randomized controlled trial, in which LDL, non-HDL, total cholesterol, and lipoprotein a (Lpa) were reduced along with an increase in HDL concentration [63]. Moreover, the activity of antioxidant enzyme superoxide dismutase (SOD) activity increased by 48%, lipid peroxidation and inflammatory protein decreased, with a lower content of malondialdehyde (MDA) and C-reactive protein (CRP) [59]. Patients with NAFLD (*n* = 50) who took phytosomal curcumin (1000 mg/day divided into two doses) for eight weeks had diminished liver fat content, and Doppler solography findings indicated an increase in hepatic vein flow with a reduction in portal vein diameter and liver volume. In addition, hepatic enzyme levels, such as aspartate aminotransferase (AST) and alanine aminotransferase (ALT), were reduced in a between group-comparison in a randomized controlled trial [62].

Anti-inflammatory and antioxidative effects of curcumin may be explained by its potential to downregulate nuclear factor-κB (NF-κB) signaling and, consequently, reduce the expression of pro-inflammatory cytokines, such as TNF-α, IL-6, and IL-1β. These cytokines have the capacity to regulate the expression of CRP in human hepatocytes [104]. Furthermore, studies demonstrated the involvement of curcumin in modulating the activity of enzymes involved in inflammatory signaling pathways, such as cicloxygenases-1 (COX-1) [105] and -2 (COX-2) [106] in an in vitro assay and human gastrointestinal epithelial cells, respectively. Also, curcumin was able to modulate arachidonic acid metabolism and its metabolites, blocking cytosolic phospholipase A2 (cPLA2), decreasing COX-2, and inhibiting catalytic activity of 5-lypoxigenase (5-LOX) in HT-29 human cells, a type of human colon cancer cell [107].

### 3.2. Resveratrol 

Resveratrol, a natural poliphenolic compound occurring naturally in nuts, berries, and the skin of grapes has demonstrated positive effects on MetS [64,65] and NAFLD [67,68] in human clinical trials. A randomized, double-blind, placebo-controlled clinical trial detected beneficial effects in metabolic parameters, such as body mass index (BMI), fat mass, waist circumference (WC), and area under the curve of insulin and total insulin secretion after supplementation with resveratrol 500 mg three times per day for 90 days in individuals with MetS (*n* = 24) [65].

An important study observed the metabolomic profile of blood, adipose tissue, skeletal muscle tissue, and urine with a high dose of resveratrol (500 mg twice per day) for four months in middle-aged males with MetS (*n* = 45) in a randomized, placebo-controlled clinical trial. Findings showed that resveratrol reduced sulfated androgen precursors, especially dehydroepiandrosterone sulfate (DHEA-S), an adrenal steroid, in blood, skeletal muscle, and adipose tissue. This reduction in sulfated androgen precursors may be due to their increase in urine [64]. These lower levels of DHE-A could be related to cardiovascular protection and may also be correlated to lower BMI as demonstrated in a previous study in which higher levels of dehydroepiandrosterone (DHEA) and DHEA-S in middle-age women were strongly associated with cardiovascular risk, and displayed a strong correlation with higher BMI and LDL levels [108]. 

Regarding the effects of resveratrol on the metabolomic profile, the metabolites of long-chain PUFAs (n-3 and n-6) increased in adipose tissue, which may be explained by the increased conversion of alpha linolenic acid (ALA) and linoleic acid influenced by reduced androgen precursors. Additionally, glycolysis, gluconeogenesis, and pyruvate metabolism were affected by the increase in important metabolites of the glycolytic pathway, such as glucose-6-phosphate (G6P), dihydroxyacetone phosphate (DHAP), 3-phosphoglycerate, and phosphoenolpyruvate (PEP) [64]. 

Other studies demonstrated that 500 mg resveratrol together with lifestyle changes for 12 weeks in a double-blinded placebo-controlled clinical trial in patients with NAFLD (*n* = 50) did not have beneficial effects on anthropometric measurements, insulin resistance markers such as glucose and insulin levels, Homeostasis Model Assessment (HOMA-IR), and Homeostasis Model Assessment of β- cell function (HOMA-β). Similar data was found for lipid levels and blood pressure. Resveratrol supplementation without lifestyle changes was able to diminish serum ALT and hepatic steatosis, which was detected by transient elastography, providing a quantitative and non-invasive evaluation of NAFLD by measuring hepatic fibrosis [67]. 

A randomized, double-blind, and placebo-controlled clinical trial demonstrated positive effects on metabolic parameters [68]. Chen et al. [68] observed that 600 mg/day resveratrol for three months in patients with NAFLD (*n* = 60) reduced insulin resistance (HOMA-IR), glucose, and total cholesterol, LDL, and liver enzymes, such as ALT and AST. Furthermore, resveratrol decreased TNF-α concentration with an increase in adiponectin levels, which are important pro-inflammatory and anti-inflammatory cytokines, respectively. Cytokeratin 18 (CK18) fragments, an intermediate protein in the liver involved in apoptosis, and fibroblast growth factor 21 (FGF-21), an important biomarker for diagnosis of NASH, were augmented in these individuals; however, after resveratrol supplementation, concentration of these proteins significantly decreased. 

Some studies did not find physiological effects in obese subjects with modest insulin resistance, which may be due to differences in time and concentration of resveratrol doses; however, the effects of resveratrol supplementation on metabolism may be due to the activation of an enzyme sirtuin-1 (SIRT1). In turn, resveratrol may have the capacity to mimic metabolic effects of caloric restriction [66]. In addition, SIRT-1 was suggested to reduce the inflammation pathway in IL-1β-induced human adipose tissue explants in vitro, reducing mRNA expression of cytokines, such as MCP-1, TNF-α, interleukin-8 (IL-8), and plasminogen activator inhibitor-1 (PAI-1), after resveratrol incubation, and increased adiponectin levels. Similar results were observed in differentiated human preadipocytes in primary cultures [109]. Other findings demonstrated that resveratrol was able to reduce pro-inflammatory cytokines, such as IL-6 and PAI-1, in TNF-α-induced atherogenic changes in 3T3-L1 adipocytes, suggesting that resveratrol has beneficial effects on metabolic profiles in human obesity [110].

### 3.3. Quercetin 

Quercetin, one of major flavonoids found in many plants, had effects in individuals with MetS [69,70,71]. A previous study observed that 150 mg quercetin per day in a double-blinded, placebo control, cross-over trial with six weeks’ treatment, separated by a five-week washout period in overweight individuals with high cardiovascular disease risk (*n* = 93), reduced systolic blood pressure (SBP) in hypertensive patients with a decrease in serum HDL concentration, whereas other lipid concentrations, such as total cholesterol, TAG, LDL:HDL, and TAG:HDL cholesterol ratios were unaltered. In addition, quercetin did not alter atherogenic oxidized LDL, TNF-α, and CRP concentrations when compared with the placebo group [69].

The same group demonstrated that not only blood pressure but also serum lipids were modified depending on different genotypes of apoliprotein E (ApoE) in individuals with MetS (*n* = 93) [71]. ApoE is a polymorphic protein that possesses three isoforms in humans, such as ApoE2, ApoE3, and ApoE4. ApoE3 is the most common isoform and is an important modulator of many stages of lipoprotein metabolism, whereas ApoE4 is strongly associated with cardiovascular disease (CVD) and high lipid levels [111,112,113,114]. In a double-blinded, placebo controlled, cross-over study, treatment with 150 mg quercetin per day for six weeks, separated with a five-week washout period in different individuals, who were separated in subgroups of ApoE3 and ApoE4, quercetin was able to decrease systolic blood pressure (SBP) in the ApoE3 subgroup without differences in ApoE4 group, increased the LDL:HDL cholesterol ratio, and reduced serum HDL and apoliprotein A1 in the ApoE4 subgroup with no differences in ApoE3 subgroup. Yet, in both subgroups, quercetin was able to reduce LDL oxidized and TNF-α levels [69]. 

Conversely, a single-center, double-blind, randomized, cross-over, placebo-controlled study was performed in individuals (*n* = 49) with different ApoE3 and ApoE4 genotypes during two eight-week treatment periods with 150 mg of quercetin per day separated by a three-week washout period. The data displayed only genotype-dependent differences in waist circumference and BMI. Moreover, genotype-independent differences were observed in some cardiovascular disease parameters, like reducing postprandial TAG and increasing HDL concentrations without differences in endothelial function. Nevertheless, quercetin increased TNF-α and diminished glutathione (GSH), suggesting slightly pro-inflammatory effects independent of genotype [71]. 

For higher BMI and waist circumference in human MetS, adipose tissue has an important role in the development of metabolic syndrome. For a better understanding of the mechanisms involved, some molecular studies are presented below. The molecular mechanisms used to understand the role of quercetin adipose tissue were observed in a study that used 3T3-L1 preadipocytes. This flavonoid was able to decrease adipogenesis with AMPK upregulation and its substrate, acetyl CoA carboxylase (ACC). Moreover, quercetin treatment of these cells induced apoptosis by involving mitogen-activated protein (MAP) kinases, especially extracellular signal-regulated kinases (ERKs), which is an important protein involved in cell proliferation, survival, and differentiation. Therefore, quercetin reduced phosphorylated-ERKs levels. Another subgroup of MAP kinases, c-Jun N-terminal kinase (JNK), a key regulator of inflammation and insulin resistance, displayed a decrease in the level of phosphorylated JNK expression after quercetin exposition [110]. These findings demonstrated the molecular mechanisms in which quercetin may be involved.

## 4. Natural Products: Fruits, Vegetables and Plants

### 4.1. Grapes

Fruits and vegetables have a variety of antioxidants and dietary fiber with important bioactive benefits. A food ingredient called wine grape pomace flour (WGPF), made from the skin and seed residues obtained from grapes during wine production, displayed high levels of phenolic compounds and dietary fiber [72]. A current randomized controlled trial study observed that 20 g of WGPF, as a food supplement in a usual diet, at lunch for 16 weeks in male subjects diagnosed with MetS (*n* = 25), was able to improve blood pressure, glycaemia, and postprandial insulin. Likewise, WGPF supplementation increased γ- and σ-tocopherol, an important antioxidant vitamin, and reduced protein damage, which contributed to reduce the oxidative stress [72]. 

However, the WGPF used in the study contained 52% fiber (7% soluble and 93% insoluble) and 4.4% extractable polyphenols with an antioxidant activity of 362.9 ORAC (µmoL TE/g dry matter), suggesting a product that contains antioxidant dietary fibers with important health benefits. Furthermore, individual compounds from WGPF were quantified and identified. These compounds included phenolic acids in which gallic acid represented 83%, flavonoids, especially catechin and catechin derivatives, represented 57.8%, flavonols 22.9%, and anthocyanin in which malvidin and malvidin derivatives represented 65%, which are the principal compounds responsible for the positive metabolic effects [72]. 

Given this information, the term antioxidant dietary fiber was conceptualized as approximately 50% of diet polyphenolic antioxidants transported along the small intestine may be linked to dietary fiber. Therefore, antioxidants are released from the fiber matrix within the colon by the action of the bacteria microbiota, resulting in bioactive metabolites and creating an antioxidant environment. The idea that antioxidants may be transported by the gastrointestinal tract may be an important action of dietary fiber [115,116,117].

In addition to WGPF, grape seed extract demonstrated some metabolic improvements for MetS, especially in blood pressure [73,74]. Grape seed extract was used in two different doses, 150 mg and 300 mg per day for four weeks, in a randomized placebo-controlled trial in adults with MetS (*n* = 27). Although the extract did not decrease lipid and glucose levels, blood pressure was reduced with the supplementation [73]. The grape seed extract used in the study was 93% phenolic compounds, such as proanthocyanidins and polymers of catechin and epicatechin [118]. Similar findings were observed in another meta-analysis randomized controlled trial in a double-blind and single-blind study. High concentrations of grape seed extract (100 to 2000 mg/day) were able to decrease blood pressure in these subjects. However, these effects were more pronounced in younger and obese individuals as well as patients with metabolic disturbances [74]. 

These important effects on blood pressure may be explained by the phenolic compounds present in grape seed extract. Previous studies demonstrated that in human umbilical vein endothelial cells (HUVECs), the endothelial oxide nitric synthase (eNOS) enzyme was activated by grape seed extract via the phosphoinositide 3-kinase (PI3k)/Akt pathway. Akt is a serine/threonine kinase that is recruited to the endothelial membrane by binding PI3K-produced phosphoinositides. Consequently, in the cellular membrane, Akt is phosphorylated and activates eNOS, resulting in nitric oxide (NO), which is an important vasodilator factor in the endothelium, promoting the vasodilator effect [118,119].

### 4.2. Green Tea

*Camelia sinensis*, known as green tea, has gained scientific recognition due to its health effects in cardiovascular disease [120]. Besides these effects, a study compared the influence of four cups per day of a green tea beverage and extract (two capsules and four cups water/day) for eight weeks in subjects with MetS (*n* = 35). Both products decreased body weight and BMI. However, only the green tea beverage was able to reduce lipid peroxidation with decreasing MDA and hydroxynonenals (HNE). Notably, the beverage displayed a decreasing trend in LDL and the LDL:HDL ratio in these subjects. One explanation of for the effects of the green tea beverage metabolism is the presence of catechins, a phenolic compound, such as epiogallocatechin-3-gallate (EGCG), epigallocatechin (EGC), epicatechin gallate (ECG), and epicatechin (EC). EGCG was the most abundant catechin in the green tea beverage and extract, which corresponded to 440 mg and 460 mg of EGC in each product, respectively. In addition to these findings, plasma-free catechins were observed in both supplemented groups after eight weeks of treatment [75].

Due to the reduction in lipid peroxidation, the same dosage and time of green tea treatment was used to evaluate serum antioxidant enzymes, such as glutathione, glutathione peroxidase, and catalase, and plasma antioxidant capacity in MetS individuals (*n* = 35). Both the green tea beverage and extract increased plasma antioxidant capacity and glutathione; however, no effects were observed on the serum levels of carotenoids, tocopherols, and enzyme activity of glutathione peroxidase and catalase. Only the green tea extract has decreased plasma iron without differences in other minerals like copper, zinc, and selenium [77].

MetS is characterized by elevated oxidative stress and impaired antioxidant status. Lipid peroxidation may cause injury to cells and cellular membranes, resulting in cell damage and death. Nonenzymatic and enzymatic antioxidant defense systems exist in the blood to scavenge reactive oxygen species (ROS) [121]. Some studies reported obesity-related abnormalities in the mineral status, in which obesity was associated to iron, zinc, and calcium deficiencies [122]; whereas other studies showed that iron has an important role in exacerbating oxidative stress in the pathophysiology of chronic disease [123]. Although green tea was able to reduce lipid peroxidation, new data demonstrated the effects of green tea extract on reducing plasma iron levels in obese subjects with MetS; however, further investigations into the biomarkers of iron absorption and plasma iron are needed to better understand the role of green tea polyphenols in modulating iron status in MetS [77]. 

Inflammation also contributes to MetS disturbances. Four cups of green tea beverage per day and green tea extract (two capsules and four cups water/day) in obese subjects with MetS (*n* = 35) did not alter inflammation biomarkers, such as IL-1β, IL-6, CRP, soluble vascular cell adhesion molecular-1 (sVCAM-1), soluble intracellular adhesion molecular-1 (sICAM-1), leptin, and the leptin: adiponectin ratio. However, both beverage and extract reduced plasma serum amyloid alpha (SAA) [76]. SAA is an apoliprotein expressed in both adipocytes and hepatocytes. This protein increased oxidative stress, decreased eNOs, promoted endothelial dysfunction, and impaired reverse cholesterol transport by HDL particles, suggesting that SAA is a predictor for cardiovascular disease in humans [124,125,126,127].

Another study observed that a green tea extract supplement containing 400 mg of EGCG had no effects on metabolic risk factors, including neither BMI, waist circumference, lipid levels, nor insulin sensitivity, insulin secretion, or glucose tolerance in a randomized controlled trial in MetS patients (*n* = 46). However, the supplementation modestly reduced the blood pressure of these subjects, suggesting an antihypertensive effect that may provide cardiovascular benefits [128]. Similar results for glucose levels was observed in another study, which showed that Mauritian green tea (one cup, three times a day before meals) over 14 weeks did not affect fasting plasma glucose, hemoglobin A1c, or glycated hemoglobin (HbA1c) outcomes in MetS subjects (*n* = 65) [78]. 

The waist:hip ratio and ALT levels were suppressed in women subjects with a reduction in blood pressure, and the antioxidant potential was increased, which was measured by the free radical-induced hemolysis assay, in both men and women after 14 weeks [78]. Furthermore, Mauritian green tea was found to have high levels of total phenols, flavonoids, and proanthocyanidins, which were represented by quercetin, kaempferol, and myricetin. Therefore, the high antioxidant potential for these patients may be explained by the presence of these bioactive compounds, which may improve the oxidative stress status in individuals with diabetes [78]. 

In patients with NAFLD (*n* = 17) in a randomized, double-blind, controlled, investigator-initiated trial, green tea was able to modify some metabolic parameters. Green tea was adjusted to 1080 mg per 700 mL and 200 mg per 700 mL catechin content, and a green tea-flavored beverage (0 mg per 700 mL catechin content) and was distributed to NAFLD patients, who consumed the drink with meals for 12 weeks. No differences were observed in BMI and body weight in all groups, but body fat mass decreased in the high-catechin concentration group with significant improvement in liver-to-spleen computer tomography (CT) attenuation ratio. However, serum ALT levels and urinary 8-isoprostane, which is an important in vivo indicator of oxidative stress, upon excretion were reduced in the high-catechin concentration group. These findings suggest that high catechin levels may be useful for the treatment of NAFLD and further investigations are necessary to determine the possible signaling mechanisms in human clinical trials [79]. 

### 4.3. Orange Juice

Orange juice (OJ) is a common drink that many people consume in many countries. However, some issues about orange juice consumption have been debated. One such issue is that some researchers proposed that a high dietary intake of 100% OJ could cause obesity and other metabolic syndromes in adults, which could be a consequence of high fructose consumption [123]. Despite this high presence of sugar in pure OJ, particular phenolic compounds and flavonoids present in OJ protected individuals from metabolic disturbances. As such, some studies investigated the correlation between OJ consumption and the improvement of metabolic syndrome parameters [80,81,82,83].

A randomized, double-blind crossover study with MetS subjects (*n* = 100) was conducted over a 12-week period with a seven-week washout period. These patients consumed OJ twice per day in 250 mL portions, made from fresh fruit enriched with normal (NPJ, 299 mg/day) and high (HPJ, 745 mg/day) polyphenol content (narirutin, didymin, and hesperedin). The results demonstrated that either NPJ or HPJ decreased BMI and waist circumference and leptin levels; however, only NPJ decreased blood pressure [74].

In terms of antioxidant effects, both NPJ and HPJ were able to reduce urinary 8-hydroxy-2′-deoxyguanosine (8-OHdG), an important DNA damage marker, and 8-isoprostane prostaglandin F2α (8-iso-PGF2α), recognized as one of the most important indexes of lipid peroxidation due to its specificity and stability. However, both products reduced erythrocyte catalase and glutathione reductase (GR) activities, whereas HPJ increased erythrocyte superoxide dismutase (SOD). Even though the glutathione peroxidase (GPX) was not affected by NPJ and HPJ, a relationship between SOD and GPX was observed, representing an increase in erythrocyte cell membrane antioxidant defense. Polyphenol content metabolites, such as hesperidin and naringenin, were observed in the urine of these subjects, confirming that these flavones have important metabolic effects [80]. 

OJ displays effects on the blood lipid profile [81]. A randomized controlled trial was performed in MetS subjects (*n* = 36) who supplemented with 250 mL per day of OJ for three months (12 weeks) without detrimental metabolic consequences at the end of supplementation. OJ demonstrated a high reduction of TAG levels in these individuals; however, OJ did not affect insulin sensitivity, circulating lipids such as total cholesterol, LDL, HDL, apoliprotein-A (Apo A1), apoliprotein-B (Apo B), or body weight. These effects were suggested to be provided by the presence of flavones in the OJ, such as the approximately 0.22 mmol of hesperidin and 0.03 mmol of narirutin that were consumed per day [81].

In addition, a prospective cohort study observed that 240 mL OJ and 5.5 g orange pomace (OPF) altered the time course of glucose and insulin responses after a high-fat meal, which reduced the postprandial glycemic and insulinemic responses in men (*n* = 36) with high cardiovascular risk. Together with these findings, OPF was able to lower non-esterified fatty acid (NEFA) concentrations circulating between 120 and 420 min after a high-fat meal, aligning with a potential impact on glucose metabolism [82]. High concentrations of NEFA were suggested to impair glucose metabolism by elevating β-oxidation in some peripheral tissues and by competing with glucose for oxidation, thus suppressing glucose metabolism through the inhibition of glucose transporter type 4 (GLUT4), leading to a reduction in peripheral glucose [129,130]. One explanation for these glycemic effects is that OPF had approximately 272 mg of total flavonoids; however, flavonoids were not uniquely responsible for these effects. The presence of fibers, since the micronization of OPF, may have released more polyphenols compared to juice made from lightly blended whole orange (WO) [82].

Furthermore, positive metabolic effects were observed in another study that reported that red-fleshed sweet orange juice (750 mL/day) for eight consecutive weeks in overweight/obese (*n* = 12/6) patients could improve the lipid profile, decreasing LDL, total cholesterol, CRP, and blood pressure. However, insulin resistance and antioxidant activity, through DPPH assay in serum, were improved in these individuals [83]. In addition to the other type of OJ that contains citrus flavonoids and carotenoids, red orange also contains lycopene, a powerful antioxidant capable of suppressing the singlet oxygen, thereby decreasing cholesterol synthesis, inflammatory responses, and preventing endothelial lesions [131,132]. The results demonstrated that red orange juice could improve vitamin C intake [83], suggesting that the antioxidant, anti-inflammatory, and DNA-protective activity of orange juice may generally be due to the synergic effect between bioactive compounds and nutrients, such as vitamin C, β-carotene, hesperidin, and cryptoxanthin [133,134,135].

### 4.4. Hibiscus

*Hibiscus sabdariffa* L. (HS) is a tropical herbal shrub belonging to the Malvaceae family, characterized by red calyces and flowers that are used in hot or cold beverages with a unique sour taste. Polyphenols, flavonoids, anthocyanin, and proanthocyanidin are the important bioactive compounds present in HS [136,137]. A factorial, randomized study evaluated the effects of HS extract powder (HSEP) alone (100 mg/day; corresponding to 1.42 mg/kg for a person weighing 70 kg) (*n* = 18), or together with a personalized diet intervention, according to NCEP ATP III, in MetS subjects (*n* = 22) for 30 days. This diet provided each individual with 30%, 55%, and 15% of their energy from fat, carbohydrate, and protein, respectively, and fiber content ranged from 20 to 30 g [84].

Therefore, the data demonstrated that HSEP alone could reduce glucose and total cholesterol levels, and increase HDL levels with an improved TAG:HDL ratio. In addition to HSEP and diet intervention, a triglyceride-lowering effect was observed in these individuals with MetS. Thus, anthocyanins were the principal bioactive compounds found in the HSEP, with 19.24 mg of anthocyanin per capsule. Delphidin and cyanidin-3-sambubiosides were the major anthocyanins identified [84].

An important double-blind, randomized clinical has observed that HS extract (HSE) (450 mg per capsule) in obese patients (*n* = 20), who have taken two HSE capsules after their meal three times per day during 12 weeks, had metabolic-regulating and hepatoprotective properties [80]. HSE not only reduced BMI, fat mass deposits, and wait-to-hip ratio, but also reduced serum free fatty acids with an improvement in hepatic steatosis in these subjects. Moreover, these effects were attributed to the polyphenol content in HSE, which was composed of 1.43% flavonoids, 2.5% anthocynins, and 1.7% phenolic acids [86].

Another study determined the influence of HSE (500 mg, once daily) for four weeks in patients with MetS (*n* = 20) in a double-blind, placebo-controlled clinical trial. The results demonstrated that HSE only displayed effects in reducing serum TAG levels and systolic blood pressure (SBP) without differences in glucose or other lipid profiles, BMI, insulin, hs-CRP, and MDA, suggesting some effects on hypertension and hypertriglyceridemia in MetS individuals, even with a lower dose and shorter time treatment compared with other studies [85]. 

### 4.5. Aloe vera 

*Aloe vera*, a well known Liliaceae family plant which resembles a cactus, is popularly used to treat burns and promote wound healing. Some studies have observed some important therapeutic properties of *Aloe vera*. The dried sap is a common remedy used as diabetes treatment in the Arabian Peninsula [138]. Due to hypoglycemic properties, a double-blind, placebo-controlled study demonstrated beneficial effects in prediabetes and metabolic syndrome patients who administrated two types of aloe products, UP780 (500 mg per capsule twice per day) (*n* = 15), which is an inner leaf gel powder standardized with 2% of aloesin, and AC592 (500 mg per capsule, once per day) (*n* = 15), which is an inner leaf gel powder [81]. Only AC592 was able to reduce total cholesterol and LDL levels compared to the placebo group, together with low glucose and fructosamine, which is a protein that indicates the quantity of glycated proteins in the plasma, indicating glycemic control. Moreover, UP780 decreased HbA1c, fructosamine, fasting glucose, insulin, and HOMA-IR. As oxidative stress may be an important contributor to diabetes complications, only UP780 reduced F2-isoprostanes, which is a prototypic biomarker of lipid oxidation. Thus, these aloe preparations may be possibly attractive adjunctive strategies to revert impaired fasting glucose and glucose intolerance. However, other studies are required to elucidate the bioactive compounds involved and other mechanisms of action may responsible for these effects [87].

### 4.6. Wild Bitter Gourd or Bitter Melon 

*Marmodica charantia* is a common tropical vegetable known as wild bitter melon or bitter gourd. Some studies performed in animal models observed some beneficial properties, such as anti-diabetic, anti-cancer, and anti-bacterial effects [135,136]. However, human clinical trials are required to evaluate the effects of wild bitter gourd on MetS. 

A preliminary open-label uncontrolled supplementation trial evaluated supplementation with wild bitter gourd (WBG) lyophilized powder (4.6 g per capsules per day) during three months in patients with MetS (*n* = 42). WBG supplementation reduced the MetS incidence ratio at the highest level at the end of the three-month supplementation. The difference remained after one month of ceasing WBG supplementation with a decrease in waist circumference. This study was a preliminary study that evaluated the effects of WBG on MetS subjects, suggesting other studies should be performed to better understand its influence on metabolism and the possible mechanisms of action [82].

### 4.7. Berberine or Barberry 

*Berberis vulgaris*, known as berberine, is an isoquinoline alkaloid found in herbs from China and India. Berberine has been used in medicines given its antimicrobial, stomachic, and hypoglycemic properties [89,90]. A randomized, double-blind, placebo-controlled clinical trial was performed in patients with MetS (*n* = 12) that received berberine trichloride (500 mg per capsule three times per day) before meals for three months. The data demonstrated that daily supplementation with berberine was able to reduce MetS with a decrease in waist circumference, SBP, TAG, and insulin secretion, with an improvement in insulin sensitivity [89]. 

The antiobesity and glucose metabolism effects of berberine may be explained by the modification of insulin secretion, adipogenesis, and glycolysis, which may inhibit mitochondrial functions, activate AMPK pathway, and increase GLUT4 and glucagon-like peptide-1 (GLP-1). The blood pressure effects, specifically vasodilator effects, provided by berberine may be due to its effects on endothelium and vascular smooth muscle cells, together with an angiotensin-converting enzyme (ACE) inhibitor effect, releasing NO by activating cyclic guanosine monophosphate (cGMP), and its antagonistic action on α1-adrenorreceptors on vasculature [139]. 

Some studies have demonstrated that anti-heat shock proteins (HSP) antibodies are predictors of risk of atherosclerosis and high HSP expression has a strong relationship with the manifestation of atherosclerosis. In the atheromatous plaque-rich region, HSP synthesis increases [140,141]. Since hypertension and dyslipidemia contribute to the production of HSPs and these anti-HSPs are novel cardiovascular disease risk factors, a study demonstrated that three capsules (200 mg of dried barberry per capsule) of berberine supplementation for six weeks in patients with MetS (*n* = 51) decreased antibodies and anti-HSPs 27 and 60, with a reduction in hs-CRP levels and an improved lipid profile. These results suggest that the effects of berberine on anti-HSP may be due to the anti-immune-modulatory effect of barberry, which may be a potential therapeutic strategy for patients with cardiovascular risk and MetS [90].

## 5. Vitamins 

### 5.1. Vitamin D

The primary physiological role of vitamin D (vit D) homeostasis is to regulate calcium and phosphorus homeostasis. Some evidence has suggested that, in addition to these roles, vit D may be important in the pathogenesis of a variety of endocrine diseases [142]. Humans may derive vit D from cutaneous synthesis and diet in the form of cholecalciferol (vit D3), and from nutritional supplements in the form of vit D3 or ergocalciferol (vit D2) [143]. 

To produce vit D after exposure to ultraviolet B (UVB) radiation, 7-dehydrocholesterol is converted to pre-vitamin D3 in the skin, which is subsequently converted into vit D3 in a heat-dependent process. After ingestion or synthesis, vit D is hydroxylated and forms 25 hydroxyvitamin D (25(OH)D2 or 25(OH)D3 in the liver, which is the principal circulating form with little biological activity. Therefore, 25(OH)D is converted by 25(OH)D-1α-hydroxylase (CYP27B1), an important enzyme in the kidneys, to bioactive hormonal metabolite 1,25 dihydroxy-vitamin D (1,25(OH)2D) or calcitriol. Thus, the primary action of this bioactive metabolite is through the nuclear vit D receptor (VDR), which heterodimerizes with the retinoid X receptor, binding to vit D responsive elements near target genes [143,144].

Finally, the active metabolite 1,25(OH)2D increases intestinal calcium absorption to promote osteoclast function and maintain calcium and phosphorus homeostasis and, consequently, promote bone health. However, several tissues in the body express VDR (vit D receptor) and CYP27B1, which may have a potential role not only in bone health and skeletal functions, but also in endocrine actions that may influence other important endocrine diseases [143,144].

A study evaluated the association of the active metabolite 1,25(OH)2D (*n* = 1048) and 25(OH)D (*n* = 2096), the circulating metabolite, in blood levels on some metabolic parameters in humans with MetS. High concentrations of both metabolite were found to be associated with a lower risk of higher concentrations of TAG and MetS, whereas higher 1,25(OH)2D concentrations were related to low HDL cholesterol. An increase in the concentration of 25(OH)D was associated with waist circumference which decreased. Thus, these findings suggested a significant correlation of active vitamin D and its components with MetS [91]. 

As vit D insufficiency is associated with MetS, a prospective, randomized, double-blind, parallel trial was conducted with the aim of evaluating the efficacy and security of different ergocalciferol (vit D2) concentrations. Given this information, 20,000 (*n* = 30) and 40,000 IU (*n* = 30) of ergocalciferol was tested in individuals with MetS for eight weeks. The highest concentration (40,000 IU) of ergocalciferol resulted in higher concentrations of 25(OH)D, the circulating form of vit D, in the serum of these patients [145].

Similar results in some metabolic parameters, in a randomized, controlled, double-blind study that used a high concentration of 50,000 IU of cholecalciferol per week for 16 weeks in MetS subjects (*n* = 80) demonstrated that the TAG concentration decreased without differences in other cardiometabolic risk factors [92]. This high concentration of vit D is safe since the upper limit is 10,000 IU per day [144]. Thus, this vit D concentration per week was able increase vit D levels for these subjects who had a vit D deficiency [92]. 

Another study demonstrated that a one-year randomized intervention with either 40,000 or 20,000 IU vit D3 (cholecalciferol) in overweight/obese subjects (*n* = 332) per week was able to reduce serum IL-6 levels, but with high concentrations of high sensitive-C reactive protein (hs-CRP) levels, without differences in insulin resistance measures and serum TNF-α levels [93]. Similar findings in insulin resistance measures were observed in another study that supplemented subjects with 40,000 (*n* = 28) and 20,000 IU vit D2 (ergocalciferol) (*n* = 28) per week for eight weeks, which demonstrated that vit D supplementation did not affect HOMA-IR or other metabolic risk factors [94]. Although vit D supplementation could not reverse insulin resistance and other metabolic parameters, the active metabolite 25(OH)D concentration increased in these patients [93,94]. However, growth hormone (GH) and the insulin-like growth factor I (IGF-I) axis (GH/IGF-I axis) were altered and lower levels of 25(OH)D metabolite were linked to measures of MetS. Therefore, the data demonstrated that vit D status was an isolated predictor of the GH-IGF-I axis. Supplementation with 20,000 IU of cholecalciferol (vit D3) (*n* = 130) per week in severely obese subjects for 12 months was able to decrease insulin-like growth factor (IGF-I) and the IGF binding protein-3 (IGFBP-3) ratio (IGF-I:IGFBP-3 ratio), which are important markers of insulin resistance, as low serum levels of IGF-I and increased levels of IGFBP-3 are related to increased waist-to-hip ratio, insulin resistance, and cardiovascular disease, whereas GH basal levels are slightly reduced in obese individuals [95,146,147].

The IGF-I/IGFBP-3 ratio is considered a reflection of free IGF-I. Increased concentrations of serum levels of IGF-I were found in obese and overweight subjects [148,149,150]. Thus, vit D supplementation decreased the IGF-I/IGFBP-3 ratio and the association between 25(OH)D and GH-IGF-I axis may indicate impaired glucose metabolism, such as the adverse effects of GF-IGF-I axis, which may be partially associated with vit D status for MetS [95].

Some data suggested that vit D insufficiency status, especially the low serum level of circulated form 25(OH)D, is associated with autoimmune diseases, cancer, and an increased risk of developing MetS and individual components of metabolic disturbances. The biochemical and metabolomic phenotyping and the influence of vit D supplementation in metabolomic metabotype for markers of MetS were reviewed in a double-blind, randomized, placebo-controlled study (*n* = 62). The circulating form, 25(OH)D, before vit D supplementation decreased, whereas 25(OH)D increased in subjects supplemented with vit D supplementation (15 µg per day) for four weeks [96]. The supplementation also reduced fasting insulin concentrations, the HOMA score, and CRP levels. However, other parameters, such as glucose, TAG, cholesterol, TNF-α, IL-6, C-peptide, leptin, adiponectin, and resistin, were not affected. To evaluate a number of different metabolic phenotypes in these apparently healthy subjects, and whether any of the individuals could be more responsive to vitamin D in terms of markers of metabolic syndrome, a cluster analysis was performed on a variety of targeted markers of metabolic syndrome, resulting in five different phenotypes. These markers indicated altered adipokine profiles, impaired fasting glucose phenotype, increased VLDL synthesis in the liver, and reduction in VLDL and LDL signals with glucose and glycerol phosphocholine. This indicates changes in lipid metabolism, suggesting a vit D responsive phenotype. These metabotype analyses highlight a potential use for vit D in nutritional research [96].

As NAFLD/NASH is a hepatic manifestation of MetS, some studies demonstrated that NAFLD patients display a vit D deficiency [151]. Supplementation with 25 µg of calcitriol, the active metabolite of vit D (1,25(OH)2D), together with a hypocaloric diet involving a reduction of 500 kcal per day, for 12 weeks in patients with NAFLD (*n* = 73) in a double-blind, randomized, controlled clinical trial, did not result in alterations of anthropometric parameters. However, calcitriol supplementation modified lipid levels, such reduced TAG and ALT and AST levels, increased HDL cholesterol, and had positive effects on insulin levels and HOMA-IR at the end of study [97].

Vit D is involved not only in bone health and skeletal function, but also in lipid and glucose metabolism, inflammation, and apoptosis. One aspect of NAFLD is insulin resistance, which is associated with lipotoxicity and oxidative stress. Therefore, this lipotoxicity of NAFLD may cause chronic hepatic inflammation [152]. Another study demonstrated that vit D is involved in the regulation of adipogenesis and regulation of NFκB transcription. Consequently, vit D may be related to inhibition of pro-inflammatory cytokines, such as TNF-α, IL-6, and IL-1β. Also, vit D may upregulate adiponectin secretion from adipocytes concomitantly with increasing GLUT-4 receptor expression, an important insulin-regulated glucose transporter in myocytes, improving insulin resistance. Yet, vit D may reduce inflammation by downregulating toll-like receptor (TLR). Different types of TLR, such as TLR-2, TLR-4, and TLR-9, are involved in inflammation [153].

Similar results related to the improvement of the degree of hepatic steatosis were demonstrated in another study that used 20,000 IU of colecalciferol/vit D3 for six months in NAFLD patients (*n* = 40). This short-term supplementation improved hepatic steatosis in the absence of concomitant weight loss [92]. Conversely, a study showed that daily supplementation with 2000 IU colecalciferol for six months did not correct any metabolic parameters or the degree of hepatic steatosis, suggesting a nonresponse of colecalciferol supplementation in NAFLD subjects (*n* = 42) [99].

### 5.2. Vitamin E

Vitamin E (vit E) is a lipid-soluble vitamin that exists in two major subgroups: tocopherol (TF), which are compounds with long saturated tails, and tocotrienol (T3), which are compounds with short unsaturated tails. These subgroups consist of four analogs: alpha (α), beta (β), gamma (γ), and delta (σ), which are differentiated by the location of methyl groups on the chromanol nucleous. Vit E, and especially α-tocopherol, is found in some food such as vegetable oils, palm oils, rice bran, olive, nuts, and grains [154]. 

The vit E nutrient is ingested with unsaturated fat-containing foods. Studies demonstrated that vit E absorption was impaired and lower levels of vit E were detected in subjects with MetS [100]. As vit E insufficiency may be associated with MetS, a study investigated vit E subgroup supplementation alone with α-TF (800 mg/day) and γ-TF (800 mg/day), and with α-TF and γ-TF combined (800 mg each per day) in subjects with MetS (*n* = 20 per group) for six weeks. γ-tocopherol together with α-tocopherol demonstrated superior positive effects on metabolism, resulting in diminished lipid peroxidation with reduced MDA and HNE levels. Inflammation also decreased with lower levels of TNF-α and hs-CRP, suggesting that vit E supplementation could improve oxidative stress and inflammation [87].

Another study showed that mixed T3 supplementation (400 mg/day) for 16 weeks in adults with MetS (*n* = 35) had beneficial effects on lipid profiles, displaying lower levels of total cholesterol and LDL with a decrease in chronic inflammations with reduced IL-6 and TNF-α [96]. Regarding the influence of vit E on NAFLD, a phase 3, multicenter, randomized, placebo-controlled, double-blind clinical trial according to the Pioglitazone versus Vitamin E versus Placebo for the Treatment of Nondiabetic Patients with Nonalcoholic Steatohepatitits (PIVENS) trial demonstrated that vit E therapy 800 IU daily (*n* = 84) displayed a considerable improvement on NASH in adults without diabetes when compared to the placebo group (*n* = 83) without differences between the group that were treated with an insulin-sensitizer, pioglitazone 30 mg daily (*n* = 80) and placebo group during 96 weeks. In addition, serum ALT and AST, hepatic steatosis and lobular inflammation were decreased in the treated groups with pioglitazone and vit E; however, there was not improvement in fibrosis scores between the groups. The results suggested that in comparison to placebo group, vit E showed superior results and also pioglitazone demonstrated some efficacy, notably on histological features of NASH [155].

A retrospective cohort study investigated the influence of vit E supplementation (α-tocopherol, 883 IU/day) for 182 days on NAFLD patients (*n* = 58). The supplementation decreased ALT levels without affecting other metabolic and anthropometric parameters [103]. As the studies that have demonstrated that vit E has positive effects on subjects with MetS and NAFLD are still scarce, further investigation is needed to evaluate its potential as a therapeutic agent for MetS and NAFLD.

## 6. Fatty Acids

Exposure to free fatty acids (FFAs) initiates inflammatory signaling and immune cell infiltration, leading to inflammation in metabolic tissues. The adipose tissue, under normal weight and conditions, is able to effectively store FFAs. However, in the obese state, the storage capacity of adipose tissue is exceeded, and the FFAs accumulate in metabolic tissues, skeletal muscle, liver, and pancreas causing lipotoxicity [156]. Accumulation of excess FFAs activates inflammatory pathways and impairs normal cell signaling in these tissues and organs, causing cellular disfunction [157]. Consequently, metabolic disorders such IR and DM2 are more likely to develop [158].

Dietary patterns significantly affect health status and MetS biomarkers. Therefore, knowing how each dietary component modulates health is necessary to achieve these benefits and avoid diseases. High fat intake has been related to inflammation [159,160,161], suggesting that the type of fat consumed was more important than the intake amount [162,163,164].

### 6.1. Monounsaturated Fatty Acids (MUFAs)

The effects of MUFA intake on health are controversial, with studies showing no direct impact on the risk of cardiovascular diseases [165]. One study suggested a marginal increase in risk of coronary events [166], and some showed a lower risk of developing these illnesses. One explanation for this dichotomy is that most studies do not address if the source of the MUFAs is vegetal or animal. In the Mediterranean diet, the primary source of MUFAs is olive oil [167]. Additionally, most studies replace saturated fatty acids (SFAs) for MUFAs, and the results could either be attributed to the benefits of MUFA consumption or the lower intake of SFAs, or both [168]. Numerous studies demonstrated the health benefits of MUFAs due to modulating the parameters of MetS and NAFLD.

For cholesterol levels, a high-MUFA diet (20% energy) reduced total cholesterol levels and LDL-c concentrations when compared with a high-SFA diet; whereas the Mediterranean diet (MUFA 21% energy) increased HDL-c concentrations and reduced the total cholesterol-HDL-c ratio more effectively when compared with a MUFA diet [169].

Insulin resistant subjects, the offspring of obese type 2 diabetic individuals, underwent three dietary periods of 28 days each in a crossover design. The first period was a diet enriched in saturated fat (SAT), the second was a diet rich in MUFA (Mediterranean diet), and the third was a diet rich in carbohydrates (CHOs). Although weight, body composition, and energy expenditure did not differ in any phase of the study, using dual-energy X-ray absorptiometry, changes in fat deposition were observed. During the in-CHO period, the fat mass was redistributed toward the abdominal depot, whereas periphery fat accumulation decreased compared with isocaloric MUFA-rich and high-SAT diets. This behavior led to increased postprandial mRNA adiponectin levels in peripheral adipose tissue and higher insulin sensitivity index values from a frequently sampled insulin-assisted intravenous glucose tolerance test in patients fed a MUFA-rich diet in comparison to a CHO-rich diet [170]. 

A study of metabolic alterations and circadian cycle demonstrated significant gene-diet interactions that could be modulated via MUFA consumption. When the MUFA intake was below 13.2% of energy intake, no differences were found in plasma glucose concentration or IR, and the opposite was true for MUFA intake above 13.2% for the carriers of minor allele rs4580704, exerting a protective effect in these gene carriers [171].

A randomized intervention evaluated the effects of dietary intervention, with and without exercise, on fat liver content. The dietary interventions were based on strategies recommended by the American Dietetic Association: a high carbohydrate (50%), high fiber, and low glycemic index diet or a reduced carbohydrate (40–45%) diet enriched with MUFAs. After eight weeks of intervention, the liver fat content significantly decreased in the MUFA (~29%) and MUFA with exercise (~24%) groups, whereas the fat liver content decreased to about 6% in the CHO/fiber group, and to about 4% in the CHO/fiber with exercise group [172].

A diet supplemented with 10 mL/day of extra virgin olive oil plus 3 g/day PUFA n-3 for 90 days resulted in a decrease in LDL-C levels, TC/HDL-C, and LDL-C/HDL-C. Extra virgin olive oil and fish oil were assumed to have a synergistic action on several MetS parameters, which could be even greater than the expected benefit with the use of an oil in isolation [173].

### 6.2. Polyunsaturated Fatty Acids (PUFAs) 

The n-3 and n-6 PUFAs are essential and they cannot be synthesized in the human body. Both essential fatty acids are metabolized to longer-chain fatty acids of 20 and 22 carbons atoms. ω-6 is metabolized to arachidonic acid (20:4n6) and n-3 is metabolized to eicosapentaenoic acid (EPA; 20:5ω3) and docosahexaenoic acid (DHA; 22:6n3). Since n-6 and n-3 are not interconvertible, are metabolically and functionally distinct, and often have opposing physiological effects, maintaining a balanced intake is important [174]. The Western diet contains excessive amounts of n-6 PUFAs and low levels of n-3 PUFAs, which leads to an unhealthy n-6/n-3 ratio of 20:1, instead of 1:1, which was consumed throughout evolution [174,175].

PUFAs can exert their MetS-improving effect by coordinating the suppression of lipid synthesis in the liver, upregulating fatty acid oxidation in the liver and skeletal muscle, and increasing body glycogen storage. To repartition these metabolic fuels, Δ-desaturase of n-6 (18:2) and n-3 (18:3) is required, with n-3 being more potent [176,177]. The intake of n-3 fatty acids partially substitutes the n-6 fatty acids in membranes of likely all cells, but especially in membranes of platelets, erythrocytes, neutrophils, monocytes, and liver cells [174].

Eicosanoids products derived from n-6 PUFAs, such as prostaglandin E2 and leukotriene B4 synthesized from arachidonic acid, are more powerful mediators of thrombosis and inflammation than similar products derived from n-3 PUFAs, such prostaglandin E3 and leukotriene B5, synthesized from EPA. In addition, an unbalanced n-6/n-3 ratio in favor of n-6 PUFA is highly prothrombotic and proinflammatory, which can contribute to trigger atherosclerosis, obesity, diabetes, and NAFLD [174,175,178,179]. Taking that into consideration, several studies assessed the effects of n-3 EPA, DHA supplementation, and the intake of foods rich in n-3 series fat, such as fish oil.

Inflammation parameters were assessed in the use of long chain n-3 fatty acids supplementation for six months (4.2 g/day). Plasmatic fatty acids levels increased significantly, but not enough to alter IR and lipolysis, as levels of basal IC palmitate, macrophage, glucose, and insulin did not differ from the placebo group [180]. 

Positive and potentially atheroprotective effects were observed after inclusion of a combination of niacin (2 g/day) and omega 3 (4 g/day) in two different studies. The first study showed additive effects on serum TG, and HDL and LDL size and buoyancy, which is consistent with a decrease in the risk of coronary events [181]. The other study showed LDL enrichment with apoE and apoA1, which may be beneficial due to the atheroprotective properties of apoE and HDL2 (a likely source of apoA1 in the LDL fraction) after 16 weeks of intervention [182]. Supplementation with only EPA also had benefits. In Japanese subjects treated with EPA (1.8 g administered daily for three months), arterial stiffness was improved, possibly through the suppression of serum amyloid A-low-density lipoprotein (SAA-LDL), an oxidized LDL (oxLDL), leading to a reduction in the frequency of cardiovascular disease development in metabolic syndrome [183].

Hypolipidemic effects were also found after enriching muffins with safflower oil (PUFA), with a reduction in triglycerides levels when compared with the group that received muffins with added MUFA (high oleic sunflower oil), both with concomitant caloric restriction of saturated fatty acids, demonstrating the importance of improving the quality of ingested lipids [184]. After a 12-week clinical intervention in adults with a MetS profile, the group whose diet was low in fat, with a high amount of complex carbohydrates (LFHCC), and n-3 PUFA supplementation (EPA and DHA at 1.4 g/day) showed improvements in MetS parameters. The main results obtained were the reduction of waist circumference, and lower blood pressure values related to hypertension and serum triglycerides, when compared with high-fat diets including both MUFA and SFA [185]. Another study evaluated the same diet: LFHCC and n-3 PUFA supplementation (1.24 g/day) for 12 weeks. An endocrine modulation due to the supplementation of this marine source was observed. The biogenesis of lipid droplets was modified, as suggested by the increase in caveolin gene expression (CAV1), a lipid droplet-related protein, and an improved fatty acid binding protein 4 (FABP4) expression, a protein involved in acid metabolism fatty acids, when compared with the same diet without n-3 PUFA supplementation [186].

n-3 PUFA mainly exerts its effect on fat reduction by extensively regulating lipid metabolism through the inhibition of lipogenesis, promotion of lipolysis, oxidation of fatty acids, and suppression of preadipocyte differentiation. The effects of n-3 PUFA on lipolysis can be modulated by perilipin and/or hormone-sensitive lipase (HSL). Perilipin recovers intracellular lipidic particles in adipocytes, and their decrease increases the access of HSL to adipocytes, hydrolyzing these fat droplets, leading to increased lipolysis. The lipase activity associated with lipoprotein lipase (LPL) is also mediated by n-3 PUFA. The LPL enzyme is located in the endothelial layer of capillaries in adipose and muscular tissues. The LPL releases fatty acids through the hydrolysis of chilomicron and VLDL-triacylglycerol, reducing their blood levels, resulting in a lipid-lowering effect [187].

Consumption of a LFHCC diet supplemented with 1.24 g/day of long-chain n-3 PUFA (1.4 eicosapentaenoic acid:1 proportion of docosahexaenoic acid) for 12 weeks improved systemic insulin sensitivity compared to a standard diet, evidenced by decreased plasma insulin, a homeostasis model assessment of insulin resistance (HOMA-IR), and nonsterified fatty acid (NEFA) concentration in subcutaneous white adipose tissue (WAT) [188]. Given this result, the n-3 PUFA supplementation exerted a modulatory effect on insulin signaling in MetS through their regulatory action on the adipose tissue gene expression profile. GAPDH, a glycolytic and gluconeogenesis enzyme, negatively affects insulin signaling through the dephosphorylation of the downstream signaling component, phosphatidylinositol-3,4,5-triphosphate [189]. n-3 PUFA supplementation may have prevented the activation of GAPDH phosphoinositide phosphatase activity in adipose tissue [188].

Another study reported improvements in the NAFDL biomarkers associated with hyperlipidemia using 4 g of fish oil supplementation for three months. The plasmatic levels of EPA, DHA, and adiponectin increased significantly, whereas the concentrations of triglyceride, total cholesterol, glucose, and apolipoprotein β and γ-glutamyl transpeptidase decreased, leading to an improvement in the metabolic aspects of NAFDL [190]. Patients with NAFDL and DM2 were supplemented using the same dosage of 4 g/day for 15–18 months of n-3 PUFAs and were evaluated on the threshold of vibration perception and microvascular cutaneous reactivity, which are indicators of early complications of DM2, insulin resistance, and obesity. The treatment resulted in a slight improvement in the threshold of vibration perception, but not associated with DM2 [191].

The inclusion of 2 g/n-3 PUFA per day indicates that levels of alanine aminotransferase, triglycerides and serum tumor necrosis factor alpha, as well as liver improved after 6 months of treatment; however, participants were included in a weight-loss program that was guided by American Heart Association (AHA) recommendations, which may have improved results, due to lifestyle changes in conjunction with supplementation [192].

Nevertheless, the supplementation of a highly purified EPA ethyl ester (EPA-E (22:5 n-3); ethyl icosapentate) at 1800 mg and 2700 mg/day dosages did not demonstrate any improvements in NASH-related histologic end points or associated markers of insulin resistance, inflammation, or hepatic injury and fibrosis [39]. Another clinical trial which performed a study with 0.945 g of n-3 PUFA (64% ALA, 16% EPA, and 21% DHA) for 6 months did not find statistical significance for several biochemical markers of liver function and glucose metabolism in patients with NASH diagnosticated [193]. The lack of beneficial effects significantly after the use of n-3 PUFA supplementation may be due to the low dosage of DHA or even its absence, since DHA is more effective than EPA in the modulation of specific markers of inflammation and blood lipids [194]. Besides, DHA is related to suppression of diet-induced steatosis, inflammation and fibrosis [195].

Isolated DHA, used as supplementation at a dosage of 3 g/day, effectively modulated the concentration of IR markers, such as triglycerides/HDL ratio (34%), plasmatic nonesterified fatty acid, and LDL-c concentration [196]. The increased dosage of DHA in the same proportion of EPA/DHA remains to be established, since most studies do not clearly specify dosage.

Vegetal origin n-3 PUFA, such as steriadonic acid (SDA; C18:4-n3), is considered a good precursor for the endogenous synthesis of n-3 PUFA, leading to EPA formation, with high Δ6-desaturase activity [197,198,199]. The determination of serum PUFAs can be used as a biomarker for MetS. hTe intake of 15–20 g of *Echium plantagineum* oil (a SDA source) increased EPA and docosapentaenoic acid (DPA) levels and reduced triglycerides [200]. Serum n-6 PUFA and Δ5-desaturase are related to a lower risk of MetS development [201]. An intake of 5 g/day for eight weeks of *Echium plantagineum* oil resulted in a higher plasmatic concentration of EPA, DPA, red blood cells (RBC), and peripheral blood mononuclear cells (PMBC) when compared with linseed oil intake [198]. However, a study with 30 g of flaxseed supplementation (about 7 g of ALA per day) in a Chinese population with metabolic syndrome risk factors significantly increased total n-3 PUFAs, ALA, EPA, and DPA, but not DHA, as no additional benefits were observed beyond the lifestyle consulting for the multiple biomarkers tested (inflammatory and endothelial markers). No significant beneficial outcome was observed by Dewell et al. [202] after ingestion of ALA (6.6 g/day) for eight weeks. This suggests that because of the low dosage of ALA ingested positive biological effects in people with MetS would not be recorded [203].

Another study [204] showed the importance of higher doses of ALA being included daily and for a long duration. One study evaluated patients randomized to an energy restricted diet enriched with ALA (approximately 3.4 g ALA/day) or a restricted energy control diet (approximately 0.9 g ALA/day). After an intervention period of 26 weeks, an improvement in the biomarkers of endothelial function, resting blood pressure (BP), and inflammation was detected in both groups. High ALA intake led to a more pronounced reduction in serum YKL-40 concentration and diastolic BP compared to the low ALA intake control diet. This result suggests a cardioprotective effect, since YKL-40, also known as human cartilage glycoprotein 39 or chitinase-3-like protein 1 is associated with endothelial dysfunction, atherosclerosis, insulin resistance, and type 2 diabetes when elevated [205]. Higher long-term doses of ALA, in conjunction with a controlled diet, can be used for patients with MetS, improving endothelial function. The intake of about 2 g per day of marine omega-3 for 12 weeks resulted in better endothelial function and arterial stiffness with a parallel anti-inflammatory effect in MetS adults [206].

When comparing supplementation of fish oil with rich-SDA botanical oil borrage (3 g/day), both enriched with n-3 PUFA, for eight weeks in adults with diabetes or MetS, the fish oil significantly lowered lipid serum profile biomarkers such as triglycerides (187.2 to 156.8 mg/dL; *p* = 0.01) and increased insulin levels (19.1 μUI/mL to 24.6 μU/mL; *p* = 0.02). However, fasting glucose levels did not result in any significant improvement. The botanical oil improved total cholesterol level (182.0 to 171.9 mg/dL; *p* = 0.05) or LDL-c (106.3 to 96.8 mg/dL; *p* = 0.04) [207]. However, IR parameters are not yet fully understood [208].

Fish intake is associated with the prevention of diseases related to MetS and NAFLD, as fish is rich in n-3 PUFA and contains high concentrations of EPA and DHA [163,209], which helps to improve biochemical parameters, such as increasing high density lipoprotein (HDL) levels and lowering triglyceride levels [210]. The effects of n-3 PUFAs in a specific ratio of EPA and DHA should be better understood if they are to be used as nutraceuticals. Their health benefits have drawn attention for the prevention and improvement of MetS.

Although several studies of patients with MetS adults have reported satisfactory results with n-3 PUFA supplementation, other studies did not observe significant improvements following intervention with this supplement. One study performed some analyses of aortic systolic blood pressure, inflammatory cytokines, red blood cell, and plasma phospholipid fatty acid profiles, after supplementation with 1.7 g/day of PUFA n-3 for four weeks, which showed no differences [199]. A second study also did not find any significant changes when evaluating the effects of n-3 PUFA (1.2 g/day) supplementation on the BP of MetS subjects consuming a low-fat, complex carbohydrate-rich diet [211]. The absence of metabolic effects with this supplementation may be due to the short time frame of the study and the absence of any diet intervention with the participants. In another study, the lack of BP-lowering effects of long-chain marine PUFA n-3 supplementation can be explained by the relatively low dose (1.2 g/day) used, since high doses (>3 g/day) of marine n-3 PUFA seems to lower blood pressure in hypertensive patients [212]. However, supplementation over a long period is key to obtaining satisfactory effects on BP when associated with the use of antihypertensives, according to a study of supplementation with 1 g of n-3 PUFA for six months, which lowered systolic blood pressure and positively modulated the blood lipid parameters [213].

In general, the introduction of unsaturated fatty acids, both MUFAs and PUFAs, and especially the latter, are effective at controlling and treating MetS and NAFDL. Notably the time required for such positive effects should be long term, with differences being reported at around 12 weeks. Supplementation should be associated with some feeding-related alteration, since it appears to be a fundamental factor in achieving better results in this group of individuals, mainly due to the large number of metabolic complications involved. Supplementation with n-3 PUFA at doses of at least 2 g daily were shown to be more effective under these conditions.

## 7. Materials and Methods 

The present review was based on results published in selected articles over a 10-year period from 2008–2017 describing clinical trials about MetS and NAFLD. The inclusion criteria were: patients (age: 18–65 years old), men together with women and human clinical trials. The exclusion criteria were: article reviews, influence of exercise, elderly, adolescents, children, and animal model studies. The key words used for the research at PubMed were: bioactive compounds metabolic syndrome human clinical trials, bioactive compounds metabolic syndrome human clinical trials, nutraceuticals metabolic syndrome human clinical trials, nutraceuticals NAFLD human clinical trials, dietary supplementation, and metabolic syndrome.

## 8. Conclusions

Nutraceuticals and dietary supplements are a promising field of study for alternative medicine, which are strategies to reduce the onset and progression of MetS and its related pathologies, such as NAFLD. Foods rich in polyphenols and phenolic compounds, in addition to vitamin D, fruits, and vegetables, are effective at reducing pro-inflammatory cytokines found in MetS, reducing their signaling pathways. These nutraceuticals had positive effects in reducing cardiovascular risks, both in biochemical parameters and by hormonal modulation, and demonstrated hepato-protective effects. In addition, the presence of phenolic compounds and catechins reduced body weight in adults, being a potential adjuvant treatment for obesity. The inclusion of fish oil in the diet, especially with EPA and DHA, improved the lipid profile, inflammatory markers, and endothelial function.

The dose, time of treatment, long-term supplementation, and new alternatives to improve the bioavailability of these dietary supplements are important issues to be evaluated. Finally, changes in lifestyle, such as diet, play a fundamental role in these results, and the introduction of these nutraceuticals may be an alternative or additional therapeutic resource with considerable potential in the treatment of MetS and its related disorders, especially NAFLD.
